# Evidence and Transparency are Needed to Develop a Frontline Health Worker mHealth Assessment Platform

**DOI:** 10.4269/ajtmh.19-0411a

**Published:** 2019-10

**Authors:** J. Mark Ansermino, Matthew O. Wiens, Niranjan Kissoon

**Affiliations:** Department of Anesthesiology, Pharmacology & Therapeutics; University of British Columbia; Vancouver, Canada; Centre for International Child Health; BC Children’s Hospital; Vancouver, Canada; E-mail: mansermino@cw.bc.ca; WalimuKampala, Uganda; Centre for International Child Health; BC Children’s Hospital; Vancouver, Canada; E-mail: matthew.wiens@cw.bc.ca; Centre for International Child Health; BC Children’s Hospital; Vancouver, Canada; Department of Pediatrics and Emergency Medicine; University of British Columbia; Vancouver, Canada; BC Children’s Hospital Research Institute; BC Children’s Hospital; Vancouver, Canada; E-mail: nkissoon@cw.bc.ca

Dear Sir,

We would like to complement Finette et al.^1^ on the progress they have made in the development of a frontline health worker mHealth assessment platform for children 2–60 months of age and for an innovative study design. Mobile health platforms such as theirs have tremendous potential to bridge the gap in skilled health-care personnel shortages in low- and middle-income countries (LMICs). However, they should be developed using rigorous evidence and a transparent process to engender confidence and foster adoption in LMICs. Thus, we are concerned about the lack of reporting transparency and detail of the clinical decision process implemented within the platform. The platform uses 42 clinical data points that are interpreted based on WHO Integrated Management of Childhood Illnesses and integrated community case management protocols and “other” evidence-based data points. The individual risk assessments are based on “physician-based logic” and “Bayesian weighting and cluster pattern analysis.” No detail or references on the processes used to produce this physician-based logic or on how this was validated are provided. Indeed, the WHO protocols were not written for machine implementation and require a significant degree of interpretation before they can be implemented within computer logic. It is, therefore, important that the logic used to implement these protocols and the integration of this logic with the physician-based logic be described. To further confound interpretation, the algorithm also seems to have been updated during the course of the study.

Patients, providers, and funders are rightly very keen to adopt new technologies to overcome the limitations such as lack of access and inadequate training. However, the allure of significant positive potential leading to this very rapid growth in medical applications should not trump a rigorous evidence-based approach to developing and adopting these new technologies. Overpromising without adequate validation is amply evidenced by the recent tragic consequences of International Business Machines Watson^2^ and should be a cautionary tale for us all. We, therefore, need to uphold rigorous processes of development, reporting,^3^ and regulation. The importance of fidelity and rigor to performance has not gone unnoticed by many authorities, including World Health Organization/International Telecommunication Union^4^ and the Food and Drug Administration in a new draft document for software as a device.^5^ We would also encourage an open, unbiased, and transparent approach as is being promoted for clinical guidelines^6^ for clinical algorithms.

We applaud and would strongly support an evidence-based, data-driven, and algorithmic approach to revolutionize the current WHO protocols and significantly improve the outcomes of children around the world. However, lack of transparency and potential bias due to personal and commercial interests has the potential to introduce confusion and mistrust between patients and providers that could derail worthy initiatives by significantly limiting their potential, tarnishing their reputation, and, hence, adversely impacting their potential. Before integrating a platform such as that of Finette and colleagues into a clinical setting, we would recommend that well-established standards for model development, transparent reporting, and validation (both internal and external) be undertaken. We will then be able to confidently harness the growing potential of computing power and machine learning and produce useful and safe tools at the bedside.
